# Enhancing Paclitaxel Efficacy with Piperine-Paclitaxel Albumin Nanoparticles in Multidrug-Resistant Triple-Negative Breast Cancer by Inhibiting P-Glycoprotein

**DOI:** 10.3390/pharmaceutics15122703

**Published:** 2023-11-30

**Authors:** Wenwen Xu, Yumeng Xiao, Liang Zheng, Mingyu Xu, Xuehua Jiang, Ling Wang

**Affiliations:** 1 Key Laboratory of Drug-Targeting and Drug Delivery System of the Education Ministry, Department of Clinical Pharmacy and Pharmacy Administration, West China School of Pharmacy, Sichuan University, Chengdu 610064, China; 2021324050039@stu.scu.edu.cn (W.X.); 2022324050036@stu.scu.edu.cn (Y.X.); frankxumy0102@gmail.com (M.X.); jxh1013@scu.edu.cn (X.J.); 2 Department of Clinical Pharmacology, The Second Affiliated Hospital of Anhui Medical University, Hefei 230601, China; zhengl@ahmu.edu.cn

**Keywords:** piperine, p-glycoprotein, paclitaxel, albumin nanoparticles, breast cancer, multidrug resistance

## Abstract

Triple-negative breast cancer (TNBC) is a highly aggressive disease with rapid progression and poor prognosis due to multidrug resistance (MDR). Piperine (PIP) shows promise as a P-gp inhibitor, capable of sensitizing chemotherapeutic drugs and exhibiting antitumor properties. This study explores the inhibitory mechanism of PIP on P-glycoprotein (P-gp) and its capacity to enhance the sensitivity of paclitaxel (PTX). We subsequently evaluated the efficacy and safety of albumin nanoparticles that co-encapsulate PTX and PIP (PP@AN). The results demonstrated that PIP enhanced the accumulation of PTX intracellularly, as determined with HPLC/MS/MS analysis. PIP was also found to increase cell sensitivity to PTX. Furthermore, we explored the inhibitory mechanism of PIP on P-gp, utilizing molecular docking simulations, RT-qPCR, and Western blot analysis. PIP appears to compete with the active paclitaxel binding site on P-gp, affecting ATPase activity and downregulating the MDR1 gene and P-gp expression. In summary, PIP could inhibit P-gp and act as a sensitizer in the treatment of TNBC with PTX. Moreover, stable and uniform PP@AN was successfully formulated, resulting in a significant increase in drug accumulation within cells as well as the downregulation of P-gp in tumors at the optimal ratio (PTX:PIP = 1:2). This led to an improvement in the antitumor effect in vivo while also reducing hepatotoxicity and hemototoxicity following chemotherapy. This study comprehensively investigated PIP’s inhibitory effect and mechanism on P-gp. We present a new approach for co-delivering PIP and PTX using albumin nanoparticles, which reduced toxicity and improved therapeutic efficacy both in vivo and in vitro.

## 1. Introduction

Malignant tumors are a major public health concern threatening human health. Among them, breast cancer is the most common cancer in women worldwide [1]. In 2020, female breast cancer’s global incidence and mortality rates were 55.9/10^5^ and 15.0/10^5^ [2], respectively. Deaths caused by breast cancer rank among the top five female malignant tumor deaths, with a mortality rate of 8.8/10^5^ [2]. Notably, triple-negative breast cancer (TNBC) accounts for 15~20% of all breast cancer cases, and is highly invasive, progresses rapidly, and has a poor prognosis due to the lack of estrogen receptors, progesterone receptors, and epidermal growth factor receptor-2 expression [3,4]. There are no specific treatment targets for these patients, and the most used treatment modality is chemotherapy [5]. Despite significant advances in cancer treatment, most patients rapidly develop drug resistance and metastasis; approximately 50% of cancers respond to chemotherapy, and over 50% rapidly develop drug resistance [6].

Multidrug resistance (MDR) refers to the simultaneous failure of multiple drugs with different pharmacological mechanisms, presenting a major obstacle to effective chemotherapy. MDR mediated by efflux transport proteins is widely studied [7,8,9]. P-gp has been intensively investigated, is encoded by the ABCB1 gene, and comprises two highly conserved nucleotide-binding domains (NBDs) and two variable transmembrane domains (TMDs), forming a transmembrane protein that opens to the inner leaflets of the lipid bilayer [10,11,12]. Due to its unique structure, P-gp accommodates many substrates, leading to the efflux of virtually all chemotherapy drugs.

Studies have shown that the prognosis of breast cancer and the efficacy of chemotherapy are related to the expression of P glycoprotein (P-gp) in tumor tissues [7,13]. Moreover, the use of chemotherapy drugs can increase the expression of P-gp. High expression of P-gp in tumor cells makes tumor cells resistant to cytotoxic drugs, thereby protecting them from the effects of chemotherapy drugs [14,15]. Consequently, the development of P-gp inhibitors assumes greater significance. However, existing P-gp inhibitors are not ideal; for example, the first-generation inhibitor verapamil [16] may increase cardiotoxicity, and the second-generation inhibitor valspodar reduced clearance of etoposide and Mitoxantrine by 64% and 60%, respectively [17]. Tariquidar is more potent and specific than the previous inhibitors but has shown unexpected toxicity in clinical trials [18]. Due to the obstacles of developing small molecule P-gp inhibitors, researchers are exploring natural products and analogs as candidate drugs that could reverse MDR in cancers.

Piperine (PIP), a piperidine alkaloid extracted from black pepper, has multiple uses, including antioxidant effects against various reactive oxygen and nitrogen species [19,20,21]. PIP demonstrates notable anticancer properties, including against breast cancer [22,23], colon cancer [24], cervical cancer, and prostate cancer [25], by promoting cell apoptosis, autophagy, and modulation of signaling pathways in various cell lines. Additionally, PIP has been found to inhibit efflux transporters [26], including by preventing the release of P-gp substrates rhodamine and calcineurin in MDR cells from MDR cell lines [27], as well as increasing the accumulation of doxorubicin and cisplatin in drug-resistant cells such as MCF-7/DOX and A-549/DDP [28]. In conclusion, PIP exhibits dual properties of an antitumor agent and a P-gp inhibitor, thereby enhancing the sensitivity of tumor cells to chemotherapy drugs. This natural compound holds significant promise for further development. However, further research is needed to fully understand PIP’s sensitizing effects and mechanism. The inhibition mechanism of P-gp can be summarized [8,29,30] as follows: (1) direct interaction with drug binding sites on P-gp, leading to competitive inhibitor that blocks substrate transport; (2) affecting the activity of ATPase and exerting a non-competitive inhibition effect; and (3) inhibiting gene and protein expression.

Moreover, the clinical application of PIP in cancer treatment is hampered by its hydrophobicity. This study simultaneously administered PTX and PIP to assess their pharmacological effects by encapsulating PTX and PIP in albumin nanoparticles. Albumin, as the most plentiful plasma protein, is an ideal carrier material due to its non-toxic, non-immunogenic, water-soluble, and biodegradable properties [31,32]. Our study employed albumin as a carrier material and co-encapsulated PIP and PTX based on nanoparticle albumin bound (Nab) technology [33] to increase drug solubility, while simultaneously delivering them to the target site to achieve synergistic anticancer effects. The prescription, fundamental characteristics, stability, and drug release of the albumin nanoparticles have been confirmed in preliminary laboratory studies [34]. Subsequently, in vivo and ex vivo experiments were conducted in this study to explore the efficiency and safety of PIP and PTX co-encapsulated in albumin nanoparticles.

In conclusion, this study has two main objectives. First, to investigate how PIP enhances the sensitivity of TNBC cells to PTX and cellular accumulation, and to elucidate PIP’s inhibition mechanism on P-gp. Second, to develop and prepare an albumin nanoparticle that can encapsulate PTX and PIP. The efficacy and safety of these nanoparticles were evaluated by in vitro and in vivo experiments.

## 2. Materials and Methods

### 2.1. Cell Culture

MDCK-MDR1 and MDCK-WT were purchased in Shanghai Zhongqiao Xinzhou and cultured with DMEM medium (Gibico, Grand Island, NY, USA); 4T1 was cultured with 1640 medium (Gibico) and purchased from the Shanghai Institute of Cell Biology, Chinese Academy of Sciences. All media contained 10% FBS (Gibico) and 1% Penicillin-streptomycin. Cells were cultured in the cell culture incubator with saturated humidity (37 °C, 5% CO_2_).

### 2.2. Cell Viability Experiments

MDCK-MDR1, MDCK-WT, and 4T1 were seeded in 96-well plates at a density of 8 × 10^3^/well for 24 h. The cell was treated with PTX (Meilunbio, Dalian, China, CAS 33069-62-4) and PIP (Meilunbio, CAS 94-62-2) dissolved in DMSO and diluted in the medium containing 1% FBS for 24 h and 48 h, respectively. Then, the medium was replaced with 200 μL 0.5 mg/mL MTT (Meilunbio, CAS 298-93-1) solution for 4 h. The MTT solution was carefully discarded and 100 μL DMSO was added to fully dissolve the crystals in a shaker at 37 °C. The inhibition rate of the drug to the cells was calculated by measuring the absorbance value at a wavelength of 490 nm.

4T1 cells were seeded in 12-well plates (5 × 10^4^/well) and treated with the drug for 24 h, then fixed with 70% ethanol. Cell cycle analysis was conducted with Propidium staining (HY-KY1071) and flow cytometry, and Crystalline Violet staining (Solarbio, Beijing, China, G1062), and via observation under a microscope.

### 2.3. PTX Accumulation Detected with HPLC-MS/MS

MDCK-MDR1, MDCK-WT, and 4T1 were seeded in 12-well plates at the density of 5 × 10^4^/well for 24 h. The cell was treated with different concentrations of PTX and PIP, which were dissolved in the medium without FBS for 15 min, 30 min, 1 h, and 2 h, respectively. Then, the medium was discarded and it was washed with PBS carefully 3 times. Next was added 100 µL RIPA with 1 mM PMSF and it was placed on ice for 30 min to collect the cell lysate containing PTX. A quantity of 10 μL of internal standard CBZ (MCE, Monmouth Junction, NJ, USA, CAS: 298-46-4) solution was added to 50 μL of the drug-containing tissue lysate sample, and 150 μL acetonitrile was added to precipitated protein. The samples were vortexed well for 5 min, then centrifuged at 14,000 rpm for 10 min. The supernatant was collected and injected into the HPLC-MS/MS instrument.

### 2.4. Western Blotting and qPCR

The total protein of 4T1 cells and liver was extracted with RIPA buffer containing 1 mM PMSF and 1 mM protease inhibitor cocktail on ice. Then, this was mixed and boiled with loading buffer, and protein concentrations were measured with the bicinchoninic acid detection kit (Beyotime, Shanghai, China). A quantity of 40 µg of the protein extract from each sample was separated via 10% sodium dodecylsulphate polyacrylamide gel electrophoresis (SDS-PAGE) and transferred to the polyvinylidene fluoride membranes (Bio-Rad, Hercules, CA, USA) and blocked in 5% skim milk buffer for 3 h. It was then incubated with P glycoprotein Polyclonal antibody (1:2000, Proteintech, Wuhan, China) overnight at 4 °C and washed with TBST 3 times, and incubated with UltraPolymer Goat anti-Rabbit IgG (H&L)-HRP (1:5000, Proteintech) for 1 h at 37 °C and washed with TBST and TBS buffer. The proteins were visualized using an electrochemiluminescence (ECL) detection kit (Thermo Scientific, Waltham, MA, USA), captured with SageCapture Software, and analyzed using ImageJ software (ImageJ 1.x).

Total RNA was extracted from 4T1 cell and mouse tumor tissue using TRNzol reagent (YEASEN, Shanghai, China). RNA was converted to DNA using the PrimeScript RT Reagent Kit, then amplified using the Hieff™ qPCR SYBR Green Master Mix (YEASEN) according to the commercial instructions. The sequences of primers are listed in the Supplementary Table (Table S1). The changes in the mRNA quantity of the genes relative to the control were calculated using the 2^−ΔΔCT^ method.

### 2.5. Immunofluorescence Analysis

After treatment, the 4T1 cells were fixed in 4% paraformaldehyde for 20 min and permeabilized with cold 75% ethanol at room temperature for 20 min. They were then blocked with TBST containing 5% BSA, incubated with P glycoprotein Polyclonal antibody (1:300, Proteintech) and Goat Anti-Rabbit IgG H&L (Alexa Fluor^®^ 488) (1:2000, Abcam, Cambridge, MA, USA). The nucleus was stained with DAPI (Beyotime) and observed under an epifluorescence microscope.

### 2.6. ATP Enzyme Activity

Evaluation of P-gp based ATPase activity was conducted using the Pgp-GloTM Kit (Promega, Madison, WI, USA). Solutions were prepared with Pgp-Glo buffer reagent according to the instructions. P-gp membrane (1.25 mg/mL) was mixed with 20 μL of Pgp-Glo buffer solution, 0.5 mM Verapamil, 0.25 mM NaVO4, and 2.5× analyte solution, and placed in a 96-well plate and incubated at 37 °C for 5 min. Another 10 μL of ATP was added and it was incubated at 37 °C for 40 min. The reaction was stopped at room temperature and 50 μL of ATP detection reagent was added at room temperature for 20 min to detect the fluorescent signal RLU using a multifunction microplate reader. The basic activity of P-gpase is calculated as ΔRLU_basal_ = RLU_NaVO4_ − RLU_NT_; the basic activity of P-gpase after drug treatment is calculated as ΔRLU_TC_ = RLU_NaVO4_ − RLU_TC_. If ΔRLUTC > ΔRLU_basal_, it indicates that the test compound is an inducer of P-gp ATPase and exhibits a stronger affinity with P-gp. Conversely, when ΔRLUTC = ΔRLU_basal_, the test compound does not impact P-gp ATPase.

### 2.7. Molecular Docking

PIP and PTX structure files were obtained on PubChem, and the P-gp protein crystal structure was obtained from the PDB (PDB: 6QEE) [10]. The water molecules were removed and the ligand molecular was extracted from the P-gp protein using Pymol. Then, the molecular docking was stimulated using AutoDOCK and visualized using Pymol.

### 2.8. Preparation of PTX@AN

Albumin nano preparations were prepared via desolvation-phacoemulsification. The PIP (1 mg) and Egg Yolk Lecithin (EV) (purity > 99%, 60 mg) were dissolved with a mixed solvent of isopropanol and ethanol, and then spun using a rotary evaporator until the organic reagent dried to obtain the PIP-EV complex. A quantity of 40 mg soybean oil, PIP-EV complex, and PTX in 250 µL chloroform was used as organic phase, which was added to 5 mL of 2% albumin aqueous solution. Ultrasound was conducted in an ice bath for 8 min (150 w, 10 s on and 10 s off). Rotary steaming was used to remove the organic reagent, before centrifuging (8000 rpm, 10 min) to remove the free drug and filter, and saving at −4 °C for subsequent detection and use.

### 2.9. Characterization of Nanoparticles

The particle size and polydispersity index (PDI) were measured via dynamic light scattering (DLS) with a NanoZS90 (Malvern Instruments Ltd., Malvern, UK). The shape of nanoparticles was characterized using transmission electron microscopy (TEM) (HITACHI HT7800, Tokyo, Japan). The molecular structure was characterized by FT-IR (PerkinElmer, spectrum2, Waltham, MA, USA). Drug loading (DL) was calculated as the amount of PTX in nanoparticles relative to the whole weight of the nanoparticles. The encapsulation efficiency (EE) was calculated as the mass ratio of entrapped PTX in nanoparticles to the theoretical amount of drug used in their preparation. PTX concentration was measured using HPLC.

### 2.10. Animal Test

In female Bacl/c mice (18–22 g), aged 5–6 weeks, 100 μL of 4T1 cell suspension was injected under the right breast fat pad of the second pair of nipples, and the number of cells seeded in each mouse was about 5 × 10^5^. When the tumor volume grew to 100 mm^3^, the mice were randomly divided into 5 groups of 8 in each group: (1) control group—injection saline; (2) PTX@AN group; (3) PP@AN 1:2 group; (4) PTX-free group, PTX dissolved with Castor oil: ethanol (1:1) solution; and (5) PP 1:2 group. The dose was 0.2 mg/20 g, qod, applied 6 times, according to the PTX concentration by tail injection.

We weighed the mice every two days and measured the volume change of the tumor using Vernier calipers during treatment. The tumor volume was calculated as follows: V_tumor_ (mm^3^) = 1/2 × L × W^2^ (L—the maximum diameter of the tumor; W—the minimum diameter of the tumor).

After the end of administration, the tumor tissue was weighed and the tumor suppression rate calculated. The organs were collected, Heme and globin staining was applied, and organs were examined for pathological changes via light microscopy. Blood was collected with a tube containing heparin and WBC, RBC, and PLT levels were detected with a blood cell analyzer.

### 2.11. Data Analyst

Microsoft Excel 2018 was used for data collection, and the experimental results were statistically analyzed using IBM SPSS Statistics 25.0 software. The mean comparison of two samples was performed using the independent sample t-test, and the data analysis and significance comparison of multiple samples was performed using single factor ANOVA. The figures were created with GraphPad Prism 9.0.

## 3. Results

### 3.1. PIP Enhances the Inhibition Effect of PTX

MDCK-MDR1 and MDCK-WT cells were used to evaluate the interaction of PIP and PTX based on P-gp, for the stable and high expression of human P-gp on MDCK-MDR1 cell. 4T1 cells were used to explore the interaction between PTX and PIP in vivo and in vitro. These were derived from BALB/c mice and have similar characteristics as human TNBC [35] and high expression of P-gp (Figure S1).

The results (Table 1) show that the IC_50_ of PTX incubated on MDCK-WT cells for 24 h and 48 h (46.11 μg/mL, 9.194 μg/mL) was significantly higher than that on MDCK-MDR1 (11.12 μg/mL, 2.528 μg/mL). The result indicated that the resistance of PTX was correlated with the high MDR1 expression on the MDCK-MDR1 cells. In addition, Table 2 shows that PIP enhanced the growth inhibitory activity in a dose-dependent manner. PIP significantly reduced the IC_50_ value of PTX, and the reverse fold of PTX on 4T1 cells was up to 4.64-fold and 9.14-fold after co-incubation with PIP for 24 h and 48 h, respectively. We then evaluated the interaction between PIP and PTX on 4T1 cells using the combination index (CI) [36]. In general, a CI value < 1 indicates synergism, =1 indicates additivity, and >1 indicates antagonism. Our analysis indicated that the combination between PTX and PIP (co-exposure) with the CI value (0.88–0.41) inhibits synergism in a concentration-dependent manner.

### 3.2. PIP Enhances the Accumulation of PTX

**Figure 1:**
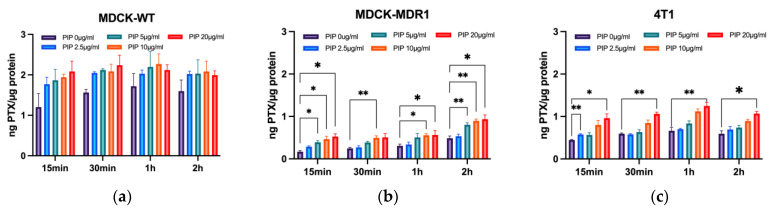
PIP could enhance the accumulation of PTX on the MDCK-MDR1 and 4T1 cells, which is related to the P-gp transporter. (**a**): The uptake of paclitaxel by MDCK-MDR1 cells incubated with or without PIP detected by HPLC-MS/MS; (**b**): the uptake of paclitaxel by MDCK-WT cells; (**c**): the accumulation of paclitaxel in 4T1 cells. Note: incubation with 5 µg/mL PTX. *n* = 3; * < 0.05, ** < 0.01.

To investigate the transport of PTX by P-gp, we detected the accumulation of PTX in cells via HPLC-MS/MS. Figure 1a shows that the content of PTX in MDCK-MDR1 cells increased significantly after incubation with PIP. The higher the concentration and the longer the time PIP was given, the greater the content of PTX that accumulated in cells. The accumulation of PTX increased to 1.85 times after co-incubation of 10 µg/mL PIP for 2 h, compared with incubation PTX alone. Figure 1b shows that the accumulation of PTX in MDCK-WT cells is much greater than that of PTX in MDCK-MDR1 cells, by about 5.6 times, which may be related to the low P-gp protein expression in MDCKII-WT cells. However, there is no significant change in MDCK-WT cells after co-incubation with PIP. Consequently, the accumulation of PTX in 4T1 cells increased significantly and up to 1.80 times after co-incubation of 20 µg/mL PIP for 2 h (Figure 1c).

Figure 1 shows that the accumulation of PTX in cells ranked as MDCK-WT > 4T1 > MDCK-MDR1. These results indicate that the content of PTX in cells was strongly contributed by P-gp and inhibited P-gp could increase the accumulation of PTX in cells. Based on these results, we can conclude that PIP can inhibit P-gp, thereby increasing PTX accumulation in cells and increasing cell sensitivity to chemotherapy drugs.

### 3.3. The Mechanism of PIP Inhibition of P-gp Function

**Figure 2:**
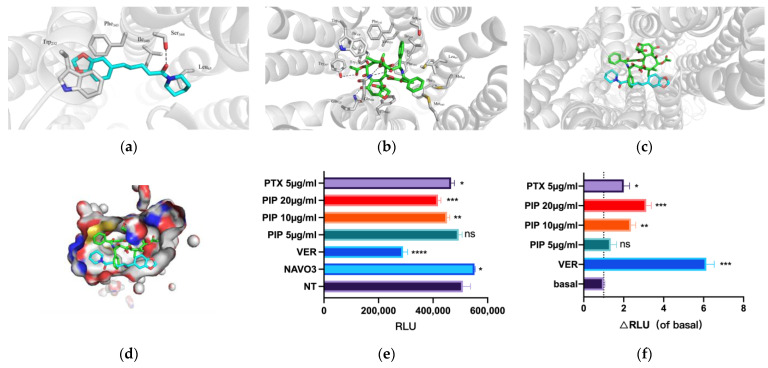
PIP had a higher affinity to human P-gp (PDB:6QEE) than PTX. (**a**,**b**): Simulation diagram of the PIP or PTX binding site on hP-gp. (Note: blue molecular-PTX; green molecular-PIP). (**c**,**d**): Simulation diagram and surface potential diagram of the interaction between PIP and PTX on hP-gp. (Note: white-hydrophobic area, blue-N, red-O, yellow-S, white-C). (**e**,**f**): The effects of PIP on P-gp ATPase activity by calculating and comparing ΔRLU. (Note: NaVO_3_—Negative control, VER—positive control). *n* = 3; * < 0.05, ** < 0.01, *** < 0.001, **** < 0.0001.

Molecular docking and the detection of p-gp ATP activity were performed to elucidate the inhibitory mechanism of PIP on P-gp. The crystal structure of the P-gp protein was obtained from the RCSB PDB (PDB: 6QEE) and was shown to bind well with PTX. Figure 2a,b depict the interaction between PIP and PTX with P-gp. Subsequently, we generated a 3D schematic diagram (Figure 2c) and a surface potential map when PIP and PTX coexisted at the drug binding site (Figure 2d). The results revealed that PIP and PTX shared numerous binding residues, including Phe343 (π-π interaction), Trp332, Ile340, Ser344, and Leu65 (hydrophobic interaction), and PIP exhibited the higher affinity for P-gp.

We quantified and compared the impact of varying concentrations of PIP on P-gp ATPase activity by assessing RLU and comparing it to that of PTX and PIP treated with ΔRLU. As depicted in Figure 2e,f, it is evident that PIP can induce ATPase activity in a concentration-dependent manner. Moreover, the capacity of PIP to induce P-gp ATPase activity at moderate and high concentrations, and its extent, surpassed that of PTX at 5 μg/mL, signifying a stronger P-gp affinity for PIP at higher concentrations.

These findings further validate the molecular docking results mentioned above, as PIP demonstrates a superior affinity for P-gp compared to PTX. Consequently, PIP can compete for the drug-binding site of PTX on P-gp at specific concentrations.

### 3.4. The Mechanism of PIP Inhibition of P-gp Expression

**Figure 3:**
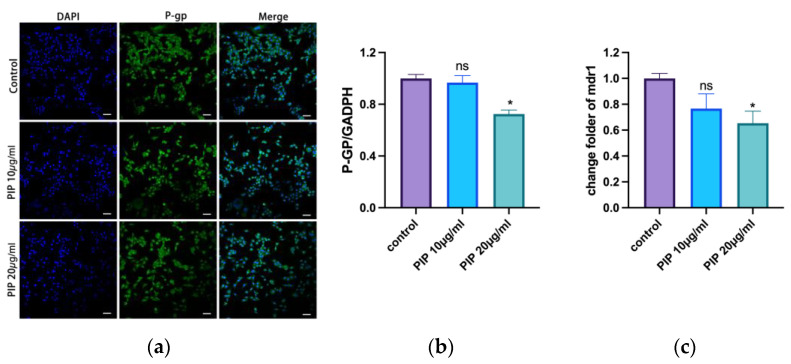
PIP could inhibit the P-gp expression and downregulate mdr1. (**a**): The effect of PIP on P-gp protein observed by IF (scale bar represents 50 μm). (**b**): Quantification of the P-gp expression caused by PIP through WB. (**c**): The effect of PIP on mdr1 gene regulation by qPCR. *n* = 3, ns > 0.5, * < 0.05, compare with control group.

In this study, immunofluorescence was used to evaluate the P-gp protein changes on the membrane surface of 4T1 cells after PIP treatment, and the expression of P-gp proteins and genes was quantitatively evaluated by WB and RT-qPCR experiments. The results showed that P-gp protein decreased as the concentration of PIP increased (Figure 3a,b), and the content of P-gp protein decreased by about 28%; with the co-incubation with 20 μg/mL PIP, the expression of the mdr1 gene also decreased (Figure 3c). The above results show that PIP can downregulate the expression of the mdr1 gene, further causing a decrease in P-gp protein.

### 3.5. Investigate the In Vitro Behavior of the PP@AN

We synthesized albumin nanoparticles that co-encapsulated PIP and PTX (PP@AN) using the desolvation-emulsification technique, as previously validated and described [34].

**Figure 4:**
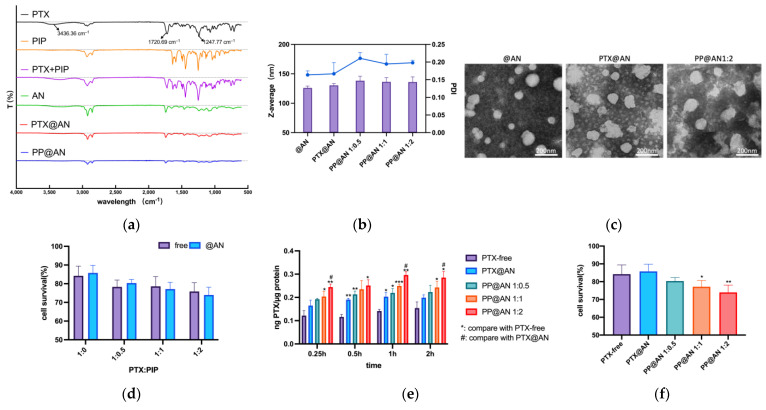

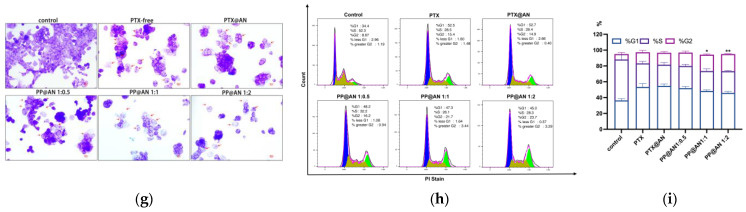
Characterization and behavior of the PP@AN in vitro. (**a**): FT-IR spectra of PTX, PIP, @AN, PTX@AN, and PP@AN; dotted line means baseline of each spectrum. (**b**): Nanoparticle size distribution diagram measured via DLS (line means PDI; bar means size). (**c**): TEM presentation of the morphology diagram. (**d**,**f**): Cell survival rate tested via MTT. (**e**): The accumulation of PTX tested by HPLC-MS/MS, h means hour. *n* = 3, * < 0.05, ** < 0.01 *** < 0.001, # < 0.05. (**g**): Crystal violet staining (red array: abnormal karyokinesis). (**h**,**i**): Cell cycle test and the count statistic. * < 0.05, ** < 0.01, compared with the cell at G2/M of control group.

The spectrum of PTX (Figure 4a, black line) showed characteristic (-N-H) stretching vibrations with H-bonded interactions (3436.36 cm^−1^), (C=O) stretching vibrations at 1720.69 cm^−1^, and ester bond stretching vibrations located at 1244 cm^−1^. However, the vibrations of PTX appear overlapped and covered by @AN, suggesting the encapsulation of nanoparticles. The DLS results showed that the nanoparticles’ size was 130 nm–136 nm, which slightly increased after drug loading. The PDI < 0.25 indicated that the nanoparticle was uniform (Figure 4b). The TEM confirmed the round shape and small size of nanoparticles. Moreover, the nanoparticles were stable when stored at 4 °C for 30 days (Figure S4). The drug loading (DL) and encapsulation efficacy (EE) were 5.2% and 92.4%, respectively, and were similar to those of a previous study [34].

Subsequently, we conducted a cell viability test between the free group and the @AN group. As the ratio of PIP increased, the @AN group exhibited a minor advantage in inhibiting cell activity. Nevertheless, Figure 4d reveals no significant difference in cell viability between the two groups when PTX:PIP was at the same ratio.

The accumulation of PTX was detected in 4T1 cells after administering to each group, as analyzed using HPLC-MS/MS. In Figure 4e, the PTX accumulation in 4T1 cells in the PTX@AN, PP@AN 1:0.5, PP@AN 1:1, and PP@AN 1:2 groups increased by 1.44, 1.56, 1.77, and 2.11 times, respectively, compared to the PTX-free group after a 1 h incubation with drugs. Notably, the cell accumulation of the albumin nanoparticle groups significantly exceeded that of free drugs. Additionally, PP@AN exhibited higher drug accumulation compared to PTX@AN, with increased intra-cellular drug accumulation at higher PIP loading ratios.

As depicted in Figure 4f, the elevated proportion of PIP in albumin nanoparticles led to a gradual decline in cell survival rates. Importantly, cell survival rates were significantly lower compared to both the PTX-free and PTX@AN group at PTX:PIP ratios of 1:1 and 1:2. Cell morphology was observed by crystal violet staining and quantified by PI staining, as shown in Figure 4g–i. Cells in the control group were morphologically intact with clear nuclei. In contrast, after PTX treatment (no PTX group and PTX@AN group), most of the cells showed a multinucleated state and the number of cells in G1 phase was significantly increased. In addition, especially in the PP@AN1:2 group, the results showed a multinucleated state and marked chromatin condensation and marginalization, as well as vacuole-like cell appearances. The number of cells entering the G2/M phase was increased significantly and cell colony numbers decreased with obvious cell debris.

The above results demonstrate that the @AN group enhances the solubility of both drugs and simultaneously delivers them into tumor cells. As a result, PIP can inhibit P-gp, increase PTX accumulation in cells, and further impede tumor cell growth, leading to increased apoptosis and decreased cell survival.

### 3.6. In Vivo Pharmacodynamics and Safety of PP@AN

**Figure 5:**
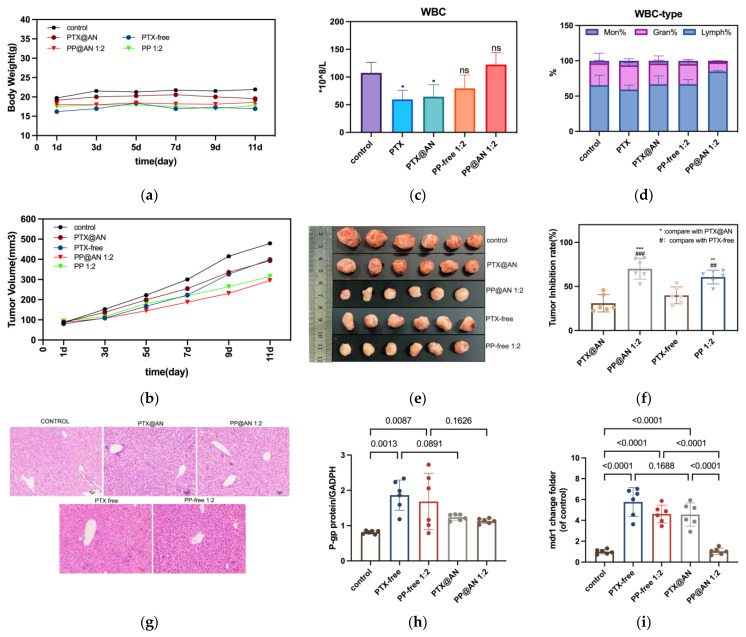
Investigated pharmacology effect and safety of PP@AN in tumor-bearing mice. Co-administration with PIP could enhance the tumor inhibition rate, and co-loaded nanoparticles showed more safety, reversing the p-gp protein and gene expression more significantly. (**a**): Body weight during the treatment. (**b**,**c**): Count of white blood cells. (**d**): The tumor volume during the treatment. (**e**): The tumor tissue gathered after treatment. (**f**): The tumor inhibition rate = tumor weight after treatment/tumor weight in control group × 100%. (**g**) The HE stanning of liver tissue (bar = 100 µm). (**h**,**i**): The P-gp protein and mdr1 gene expression of tumor tissue. *n* = 6. ns > 0.05, * < 0.05, ** < 0.01 *** < 0.001, ## < 0.01, ### < 0.001.

Pharmacodynamic studies were subsequently performed on mice with tumors to evaluate the safety and effectiveness of PP@AN. Afterwards, the antitumor effects of each group were analyzed (Figure S5 and Figure 5b). In the @AN group, with an increase in the ratio of PTX:PIP, the tumor weight decreased gradually along with a gradual increase in the tumor inhibition rate. Additionally, the co-administration group demonstrated significantly better antitumor effects compared to the PTX@AN group. However, co-administering the free drug group resulted in an increase in tumor inhibition rate, but no correlation was observed between the PTX:PIP ratio and the outcome. This could be attributed to the non-specific distribution of the free drug in vivo. Consequently, we only analyzed and depicted the PP1:2 co-administration group in the subsequent analysis.

Throughout the entire administration period, the mice in each group exhibited healthy growth, and there were no statistically significant differences in body weight (Figure 5a). The tail blood vessels in the @AN administration group remained in good condition. In contrast, the tail blood vessels in the free drug administration group became thinner, exhibited increased permeability, and were more prone to drug leakage after 4 to 5 administrations.

Figure 5c illustrates that PTX treatment decreases the white blood cell (WBC) count in the blood. The WBC count reduction is comparable between the PTX-free and PTX@AN groups, while the PTX@AN1:2 group returned to normal white blood cell levels. Nonetheless, there were alterations in WBC types (Figure 5d), with a notably higher proportion of lymphocytes (Lymph%) in the PTX@AN1:2 group compared to the control group. Conversely, the proportion of neutrophils (Gran%) was relatively reduced, indicating that drug-loaded albumin nanoparticles could activate the body’s immune system, increasing lymphocyte count. There was no difference in platelet counts between groups (Figure S6).

After the treatment concluded, we gathered and weighed the tumors of all mice groups and then calculated the tumor inhibition rate (Figure 5e,f). The tumor inhibition rate in the PP@AN1:2 group was 2.26 times higher than that in the PTX@AN group, and exceeded that in the PTX-free group. On the other hand, the PP-free 1:2 group exhibited a rise in tumor suppression rate, although the statistical difference was not significant.

HE stanning results of various organs in mice (Figure S7) revealed that different treatments did not significantly affect the heart, kidney, spleen, or lung tissues. However, notable changes were observed in the liver tissue (Figure 5g). These findings indicated that co-administrated free drugs might exacerbate liver damage. However, in the @AN group, liver cell damage was relatively mild, and such damage can be reversed upon the inclusion of a specific dose of PIP.

Lastly, we assessed the expression of the mdr1 gene and P-gp protein in tumor tissue, and the results are presented in Figure 5h,i. The mdr1 gene expression in tumor tissue was upregulated following PTX treatment alone, with approximately a 4-fold and 6-fold increase in mdr1 gene expression observed in the PTX@AN and PTX-free groups, respectively, compared to the control group. A similar pattern was observed in the changes in P-gp protein content, with the PTX-free group exhibiting significantly higher P-gp protein content than the control group. The PTX@AN group also showed higher protein content, although this difference was insignificant.

Furthermore, our results suggest that co-administration with PIP can reverse the chemotherapy-induced increase in mdr1 expression and the decrease in protein content. The PP@AN1:2 group exhibited a significant decrease in mdr1 gene expression compared to the PTX@AN group (*p* < 0.001). Conversely, the PP-free1:2 group displayed a decrease in mdr1 gene expression compared to the PTX-free group, although this difference was not statistically significant. Regarding changes in P-gp protein content, while the protein content decreased with the combined use of PIP, there was no statistical difference compared to PTX alone.

## 4. Discussion

Resistance to chemotherapy drugs in TNBC is an urgent problem that needs to be solved. More studies have found that PIP can be used as a chemotherapy sensitizer. Li et al. found that 50 μM of PIP could reverse the resistance of MCF-7/DOX and A-549/DDP to doxorubicin, with reversal resistance factors of 32.16 and 14.14 times, respectively [37]. In addition, PIP itself also has certain antitumor effects. Lai et al. found that PIP inhibits the growth of 4T1 cells in a time- and dose-dependent manner, with IC_50_ at 48 h of approximately 105 ± 1.08 μmol/L [23]. Li et al. found that the accumulation of docetaxel in MDCK-MDR1 cells is significantly enhanced in the presence of PIP [26]. A similar effect was shown in our study, under the action of PIP at 10 and 20 μg/mL, with both MDCK-MDR1 and 4T1 cells showing increased sensitivity to PTX, increasing by up to 16.3 times. At a higher PIP concentration (40 μg/mL) for 48 h, PIP exhibited a significant antitumor effect and could exert an antitumor effect together with PTX.

PIP is a potential inhibitor of P-gp due to its impact on multiple aspects of P-gp. Our findings indicate that PIP has higher affinity for the P-gp binding site and can preferentially occupy it (Figure 2c,d). The expulsion of subsequent substrate requires a conformational change in P-gp, which is coupled to ATP hydrolysis by ATPase [12]. PIP induces the ATPase activity in a concentration-dependent manner (Figure 2f). We speculated that a concentration of 5 ug/mL of PIP may only occupy the P-gp binding site without providing a sufficient trigger for ATPase hydrolysis, so that 5 ug/mL of PTX promotes higher RLU variations. Simultaneously, PIP can also directly induce the MDR1 gene to downregulate P-gp expression and inhibit P-gp from exerting a sensitization effect on PTX from multiple aspects. However, the limitation of the software’s inherent functionality restricted our ability to simulate the interaction characteristics of only one PIP molecule with PTX at P-gp. Based on the results illustrated in Figure 1, it is probable that an elevated concentration of PIP increases cellular sensitivity to PTX, and that PIP may act on multiple molecules concurrently. Nevertheless, further analyses of protein crystals are necessary to precisely elucidate the exact number of PIP molecules required to fully occupy the binding site of P-gp for PTX.

It can be concluded that PIP is a superior sensitizer for PTX in treating TNBC. However, PIP’s use in chemotherapy has been limited due to its poor water solubility. It is imperative to identify a way to improve solubility for both drugs, as well as enable their simultaneous delivery to the tumor. Burande et al. [38] reported the combination of PTX and PIP in liposomes for the treatment of TNBC, which indicated that the combination of the two drugs augmented the cytotoxicity of PTX, exhibiting a synergistic anticancer effect of PIP. In our study, we utilized albumin as a carrier to synergistically deliver PIP and PTX and extensively examined the mechanism and synergistic effect of PIP. Albumin’s ability to bind to cell surface glycoprotein receptors (gp60) makes it a particularly suitable carrier for targeted drug delivery in oncology [39]. Enhancing the specificity of delivery to the tumor site is an effective strategy to improve the efficacy of P-gp inhibitors while reducing toxicity.

The nanoparticle’s size was less than 150 nm in our study, which facilitated precise delivery of two drugs into tumor tissues through the EPR effect [40,41]. Upon entering the tumor cells, both drugs were released simultaneously. PIP effectively bound to P-gp, further preventing PTX efflux and producing a synergistic effect. The cell uptake results (Figure 4e) demonstrated that PTX@AN accumulated more in cells compared to PTX-free, and the combination of PIP resulted in greater cytotoxicity. Figure 5h,i demonstrate a significant increase in P-gp and mdr1 expression in the tumor tissues of the PTX-free group, so we assumed that long-term administration would further stimulate the expression of exocytosis transporters in the tumor, which might lead to therapeutic failure. However, PIP in PP@AN reduced P-gp expression in tumor tissues significantly compared to the PP-free group, which confirmed the important role of PP@AN in reversing tumor drug resistance. Furthermore, PP@AN demonstrated a superior safety profile compared to the PP-free group, although the antitumor effect was only slightly better. The shorter treatment period in this study could account for this result.

This approach achieved temporal colocalization of drugs and mitigates the toxicity problems associated with non-specific distribution. Concomitant administration of chemotherapeutic agents and P-gp inhibitors overcomes P-gp-mediated substrate excretion and ensures the desired therapeutic effect. This pharmaceutical delivery technique can also reduce the need for repeated drug administrations, thereby enhancing clinical patient adherence. Such an approach holds significant clinical relevance.

The study targeted the highly expressed P-gp in the tumor site, investigating PIP’s inhibitory effect on P-gp and its sensitizing effect on PTX. To enhance their pharmacological effects, PIP and PTX were co-delivered through albumin nanoparticles. Although some studies have shown that PIP has a certain inhibitory activity on the CYP450 enzyme [42], and PTX is the metabolic substrate of the CYP450 enzyme [43], further pharmacokinetic experiments can be conducted to investigate the effects of nanoparticles and PIP on the blood concentration and distribution of PTX, and to further investigate the drug–drug interactions between PIP and PTX based on the CYP450 enzyme.

## 5. Conclusions

The study’s conclusion indicates that PIP may enhance PTX accumulation and sensitivity in cells. It is proposed that this effect is achieved through competition for paclitaxel’s active binding site on P-gp, resulting in ATPase activity changes and downregulation of the MDR1 gene and P-gp expression. The present study developed a stable, homogeneous, and slow-release albumin nanoparticle that simultaneously carried PIP and PTX to tumor tissue. Through inhibiting P-gp expression and activity, PIP may enhance PTX accumulation and sensitivity in cells. This delivery system exhibited significantly heightened drug uptake in cells and superior antitumor efficacy. Moreover, it notably mitigated hepatotoxicity and hematotoxicity when administered to tumor-bearing mice. The study presented a new case using the biologically active natural compound in a skillfully designed co-delivery system to effectively enhance tumor chemotherapy and overcome drug resistance.

## Figures and Tables

**Table 1:** Combination index (CI) at the IC_50_ of PTX combined with PIP on MDCK-MDR1cells. (Mean ± SD).

Time	Cell Type	Group	IC_50_ (µg/mL)	Reverse Fold	CI	Interpretation *
24 h	MDCK-WT	PTX	11.12			
	MDCK-MDR1	PTX	46.11			
		PTX+PIP 10 µg/mL	32.24	1.43	0.80 ± 0.23	+ +
		PTX+PIP 20 µg/mL	19.31	2.39	1.06 ± 0.17	−
		PTX+PIP 40 µg/mL	5.002	9.22	1.06 ± 0.13	−
48 h	MDCK-WT	PTX	2.528			
	MDCK-MDR1	PTX	9.194			
		PTX+PIP 10 µg/mL	2.496	3.68	0.78 ± 0.38	+ +
		PTX+PIP 20 µg/mL	0.5642	16.30	0.75 ± 0.30	+ +
		PTX+PIP 40 µg/mL	0.08938		0.62 ± 0.09	+ + +

* 0.3 < CI < 0.7 means synergism (+ + +), 0.7–0.85 moderate synergism (+ +), 0.9–1.1 nearly additive (−).

**Table 2:** Combination index (CI) at the IC_50_ of PTX combined with PIP on 4T1 cells. (Mean ± SD).

Time	Group	IC_50_ (µg/mL)	Reverse Fold	CI	Interpretation *
4T1 24 h	PTX	38.62			
	PTX+PIP 10 µg/mL	28.66	1.35	0.88 ± 0.26	+
	PTX+PIP 20 µg/mL	16.21	2.38	0.65 ± 0.14	+ + +
	PTX+PIP 40 µg/mL	8.326	4.64	0.62 ± 0.21	+ + +
4T1 48 h	PTX	16.52			
	PTX+PIP 10 µg/mL	4.957	3.34	0.62 ± 0.34	+ + +
	PTX+PIP 20 µg/mL	1.808	9.14	0.53 ± 0.19	+ + +
	PTX+PIP 40 µg/mL	0.04843		0.41 ± 0.14	+ + +

* 0.3 < CI < 0.7 means synergism (+ + +), 0.85–0.90 slight synergism (+).

## Data Availability

The data presented in this study are available in supplementary material.

## Supplementary Materials

The following supporting information can be downloaded at: https://www.mdpi.com/article/10.3390/pharmaceutics15122703/s1, Table S1: The sequences of primers use for qPCR; Figure S1: The expression of mdr1 in MCF7 cell and 4T1 cells; Figure S2: The stability of encapsulation rate and size; Figure S3: The picture and weight of tumor tissue after administration; Figure S4: The counts of red blood cells and platelets; Figure S5. (a)The tumor tissue of mice after administration. (b) The tumor weight of each group after administration; Figure S6. The counts of Red Blood Cell (a) and Platelet (b); Figure S7. The HE stain of heart, kidney, lung and spleen.
