# Supplementary material for: Enhancing Paclitaxel Efficacy with Piperine-Paclitaxel Albumin Nanoparticles in Multidrug-Resistant Triple-Negative Breast Cancer by Inhibiting P-Glycoprotein
Source: Pharmaceutics. 2023 Nov 30;15(12):2703. doi: 10.3390/pharmaceutics15122703 (PMC10747290; doi:10.3390/pharmaceutics15122703)
Supplement: Supplementary file 1 [file pharmaceutics-15-02703-s001.zip › pharmaceutics-2686121-supplementary.pdf]

Supplementary:

Enhancing Paclitaxel Efficacy with Piperine-Paclitaxel Albumin Nanoparticles in Multidrug-Resistant Triple-negative Breast Cancer by Inhibiting P- glycoprotein

Wenwen Xu[1], Yumeng Xiao[1], Liang Zheng[2], Mingyu Xu[1], Xuehua Jiang[1], Ling Wang[1,*]

[1] Key Laboratory of Drug-Targeting and Drug Delivery System of the Education Ministry, Department of Clinical Pharmacy and Pharmacy Administration, West China School of Pharmacy, Sichuan University, Chengdu, 610064, China
[2] Department of Clinical Pharmacology, The Second Affiliated Hospital of Anhui Medical University, Hefei, 230601, China
* Correspondence: wlin_scu@scu.edu.cn

Table S1 The sequences of primers use for qPCR.

Oligo name	Source	Sequence（5'-3'）
MDR1-F	Human	TGACTACCAGGCTCGCCAATGAT
MDR1-R	Human	TGTGCCACCAAGTAGGCTCCAAA
GAPDH-F	Human	AGAAGGCTGGGGCTCATTTG
GAPDH-R	Human	AGGGGCCATCCACAGTCTTC
Mdr1-F	Mice	GTGGGGGACAGAAACAGAGA
Mdr1-R	Mice	GAACGGTAGACAAGCGATGAG
Gapdh-F	Mice	CAAGGCTGTGGGCAAGGTCA
Gapdh-R	Mice	AGGTGGAAGAGTGGGAGTTGCTG

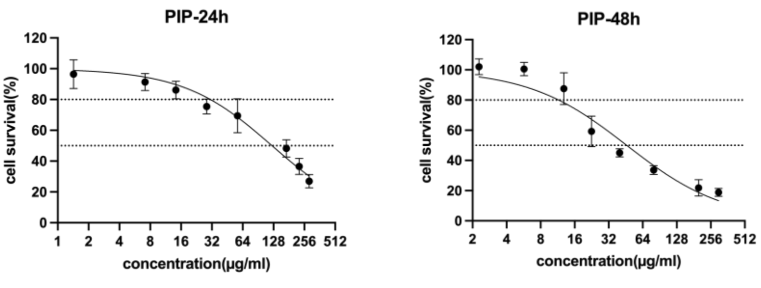

(**a**) (**b**)

Figure S1. The cell survival of PIP treated with 4T1 cells for 24 h(a) and 48 h(b) respectively.

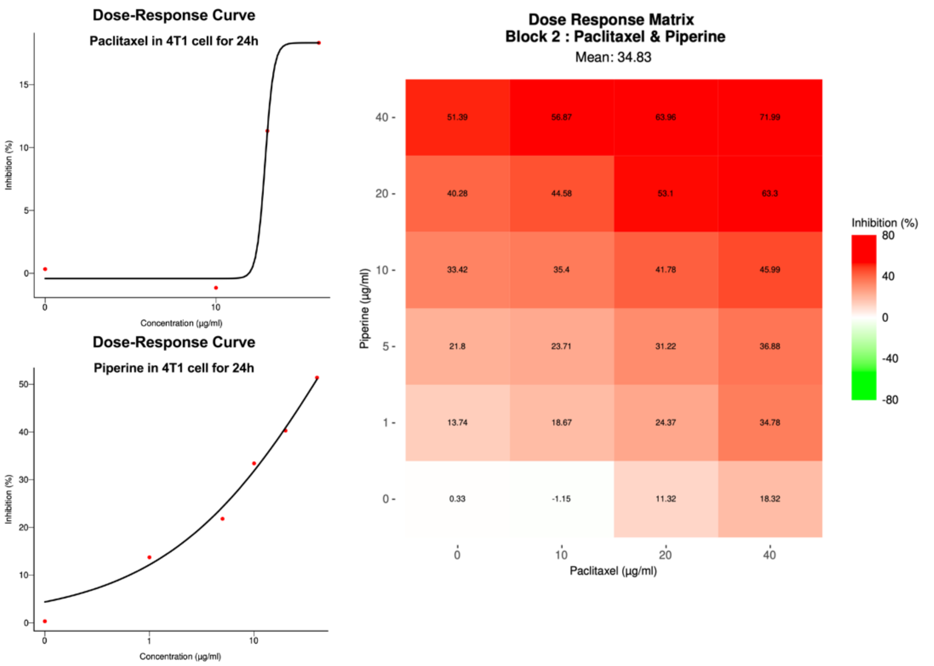

(**a**)

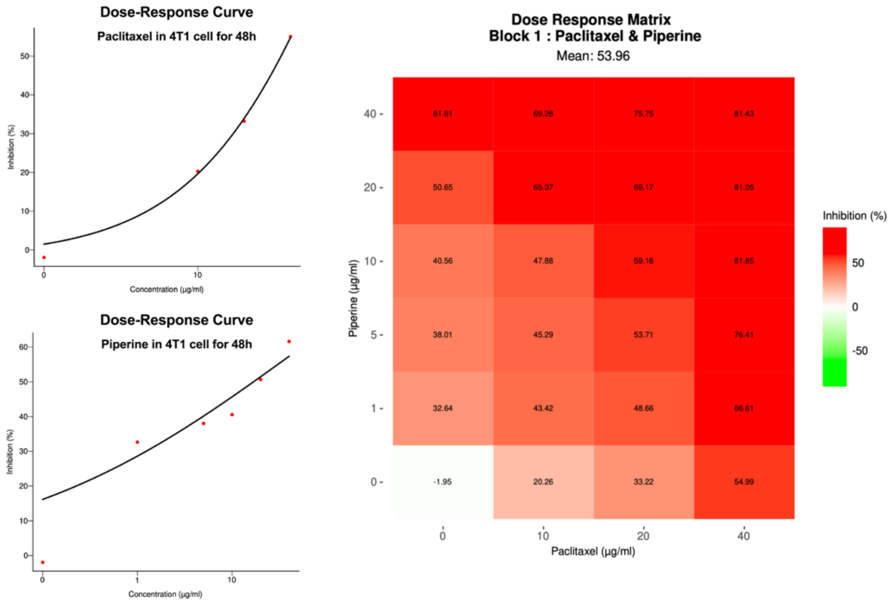

(b)

Figure S2. The does-response curve of PTX and PIP, and the dose-response matrix between PTX and PIP was draw by SynergyFinder+ (https://synergyfinder.org). （**a**）treatment on 4T1 cell for 24h. **(b)** treatment on 4T1 cell for 24h.

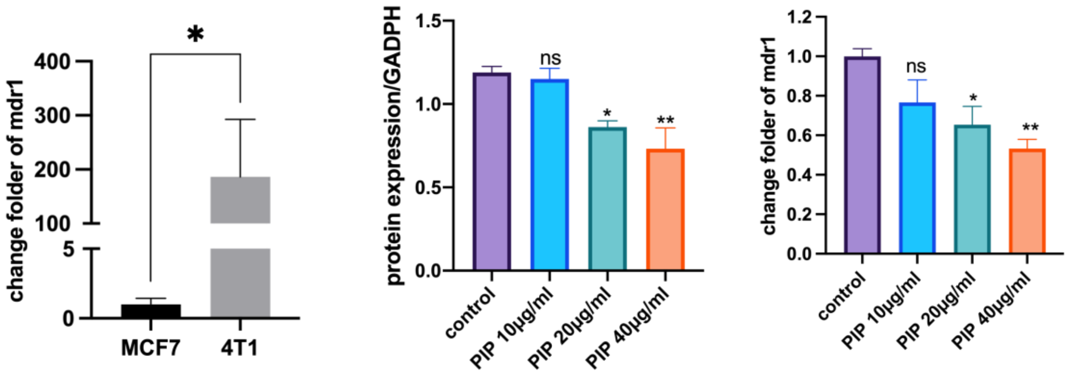

(a) (b) （c）

Figure S3. （a）The expression of mdr1 in MCF7 cell and 4T1 cells. (b): Quantified the P-gp expression caused by PIP through WB. **(c)**: the effect of PIP on mdr1 gene regulation by qPCR. n=3, *<0.05, compare with control group.

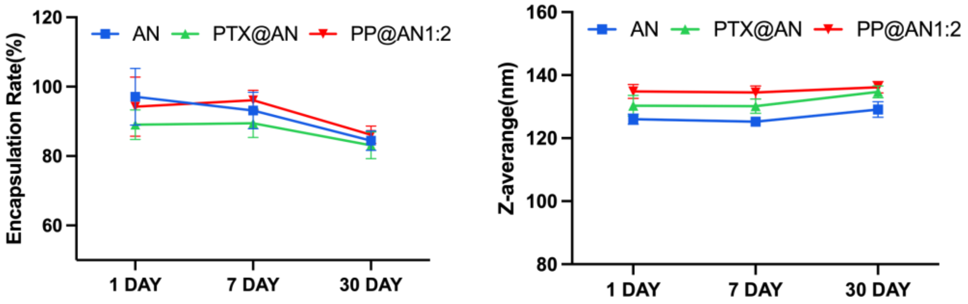

(a) (b)

Figure S4. The Stability of encapsulation rate (a) and size (b).

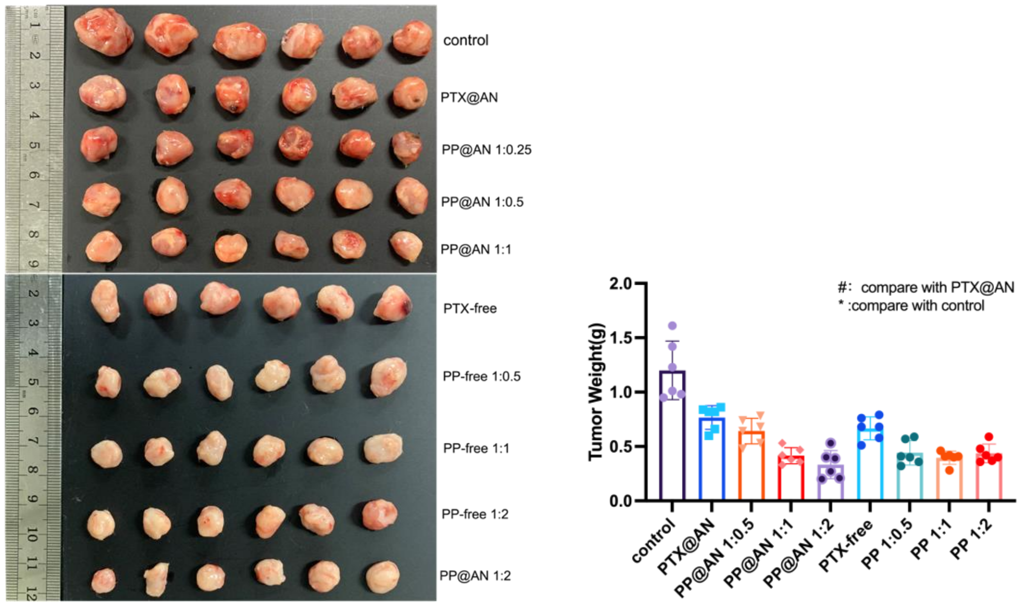

(**a**) (**b**)

Figure S5. (a) The tumor tissue of mice after administration. **(b)** The tumor weight of each group after administration.

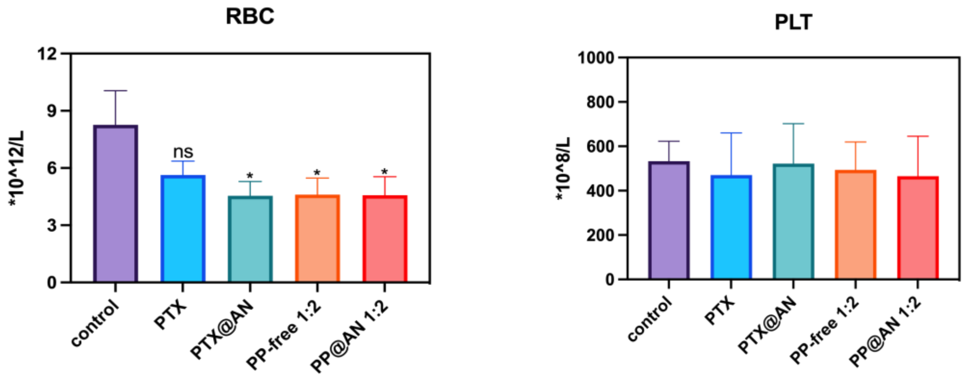

(**a**) (**b**)

Figure S6. The counts of Red Blood Cell **(a)** and Platelet **(b).**

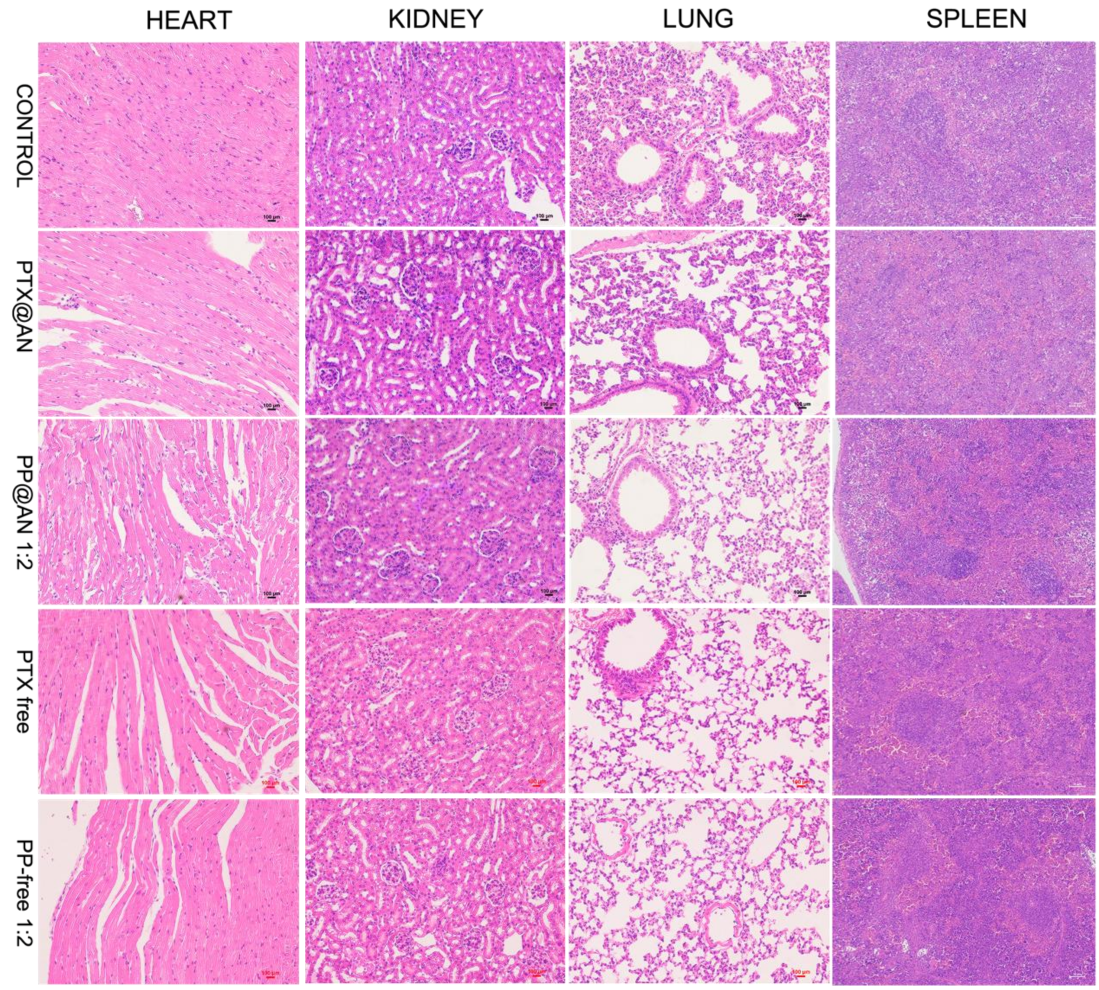

Figure S7. The HE stain of heart, kidney, lung and spleen**.**
